# Exploring global climate intervention experiments: sociotechnical promises, innovation dynamics, and perceived co-impacts across 20 projects and pilots

**DOI:** 10.1007/s11625-025-01696-6

**Published:** 2025-06-05

**Authors:** Benjamin K. Sovacool, Chad M. Baum, Livia Fritz, Sean Low

**Affiliations:** 1 Department of Earth and Environment, Boston University, Boston, USA; 2 Institute for Global Sustainability, Boston University, Boston, USA; 3 Department of Business Development and Technology, Aarhus University, Århus, Denmark; 4 Bennett Institute for Innovation and Policy Acceleration, University of Sussex Business School, Falmer, UK; 5 Wageningen University and Research, Wageningen, Netherlands; 6 Science Policy Research Unit (SPRU), University of Sussex, Jubilee Building, Room 367, Falmer, East Sussex BN1 9SL UK

**Keywords:** Carbon dioxide removal, Geoengineering, Negative emissions, Radical climate options, Climate policy, Solar radiation modification

## Abstract

Using techniques commonly applied in participatory action research and ethnography, we examine 20 specific cases of experimentation for a selection of carbon removal and solar radiation modification interventions. These experiments include engineering-based approaches such as stratospheric aerosol injection, cloud brightening, carbon–neutral cement, biochar, direct air capture, and enhanced rock weathering alongside ecosystems-based approaches such as afforestation, seagrass restoration, and coral reef protection. Based on extensive original research of these 20 experimental projects—including 118 semi-structured research interviews and naturalistic site-based observation—we explore four questions. Firstly, what are the actor coalitions surrounding each experiment? Secondly, what promises and expectations do those actors generate? Thirdly, what innovation dynamics and styles are emergent and evident here? Finally, what perceived co-impacts are expected (by actors) to occur with widespread prospective deployment? Answering these questions in our empirical study offers insights into energy, climate, and climate intervention research, given that these experiments involve some of the most powerful and dominant actor coalitions, are supported by large amounts of climate finance investment, and will undoubtedly shape future deliberations over climate policy and technology deployment.

## Introduction

Given the inadequacy of climate change mitigation efforts (Stoddard et al. 2021), coupled with perennial underinvestment in climate adaptation efforts (Fieldman 2011; Tall et al. 2021), some climate change actors are turning to consider, if not embrace, more radical interventions (Morrison et al 2022). Among these are carbon dioxide removal (CDR) or negative emissions options that seek to capture and permanently store carbon from the atmosphere (Low et al. 2022; Sovacool et al. 2023a), or regional and global proposals to reduce temperature and cool the Earth through solar radiation modification (Baum et al. 2024; SAPEA 2024; Baiman et al. 2024). Both CDR and solar radiation modification could have a profound and lasting impact not only on climate change policy deliberations, but also on community well-being and ecosystem vitality (Anderson and Peters 2016; Carton et al. 2020; Aldy et al. 2021; MacMartin et al. 2022; Baresi et al. 2025).

In this study, we focus on 20 cases of experimentation for a selection of carbon dioxide removal and solar radiation management interventions (see Sect. “Research design and analytical rubric”). These sites are critically important to the development of these nascent innovations, given that they (a) involve foundational, first-mover actors and coalitions, (b) direct large amounts of climate-related finance and innovation funding, and (c) represent loci of innovation, patenting, and rapid technological change (Kremer 2020; Acemoglu et al. 2011; Thomke 2003).

Experimentation has been regarded by policymakers, inventors, and entrepreneurs as an essential tool by which nation states and firms promote transitions toward sustainability, particularly by creating protective spaces for creative innovations to develop (Kivimaa et al. 2017). As Wang and Bai (2022: 53) recently opined, “[d]espite a growing body of literature on how to broaden urban sustainability experiments, the research community still lacks a comprehensive understanding of the factors that enable and obstruct real-world applications.”

At the same time, promissory efforts by first-mover innovators to accelerate visibility, investment, and development in nascent technologies can lead to false expectations, perverse outcomes in social and environmental impacts and, if relied on too significantly, provide the foundation for ill-formed policy. Sites of experiments and trials, whether in the laboratory or the field, are therefore a unique unit of analysis for researchers wishing to better comprehend the organizational contexts where innovation unfolds, and the drivers that shape it (Hauser et al. 2017) and the limitations they face. Sect. "Results and discussion" applies an analytical rubric to these 20 cases consisting of sociotechnical actors (Sect. “Actors, stakeholders and actor coalitions”), promises (Sect. “Promises and requirements”), innovation dynamics and styles (Sect. “Innovation dynamics and styles”), and perceived co-impacts (Sect. “Perceived co-impacts”).

## 2. Research design and analytical rubric

The next section presents our case study selection and data collection process, and the following section introduces the analytical rubric for our qualitative comparative case analysis.

### Case study selection and data collection

**Fig. 1:**
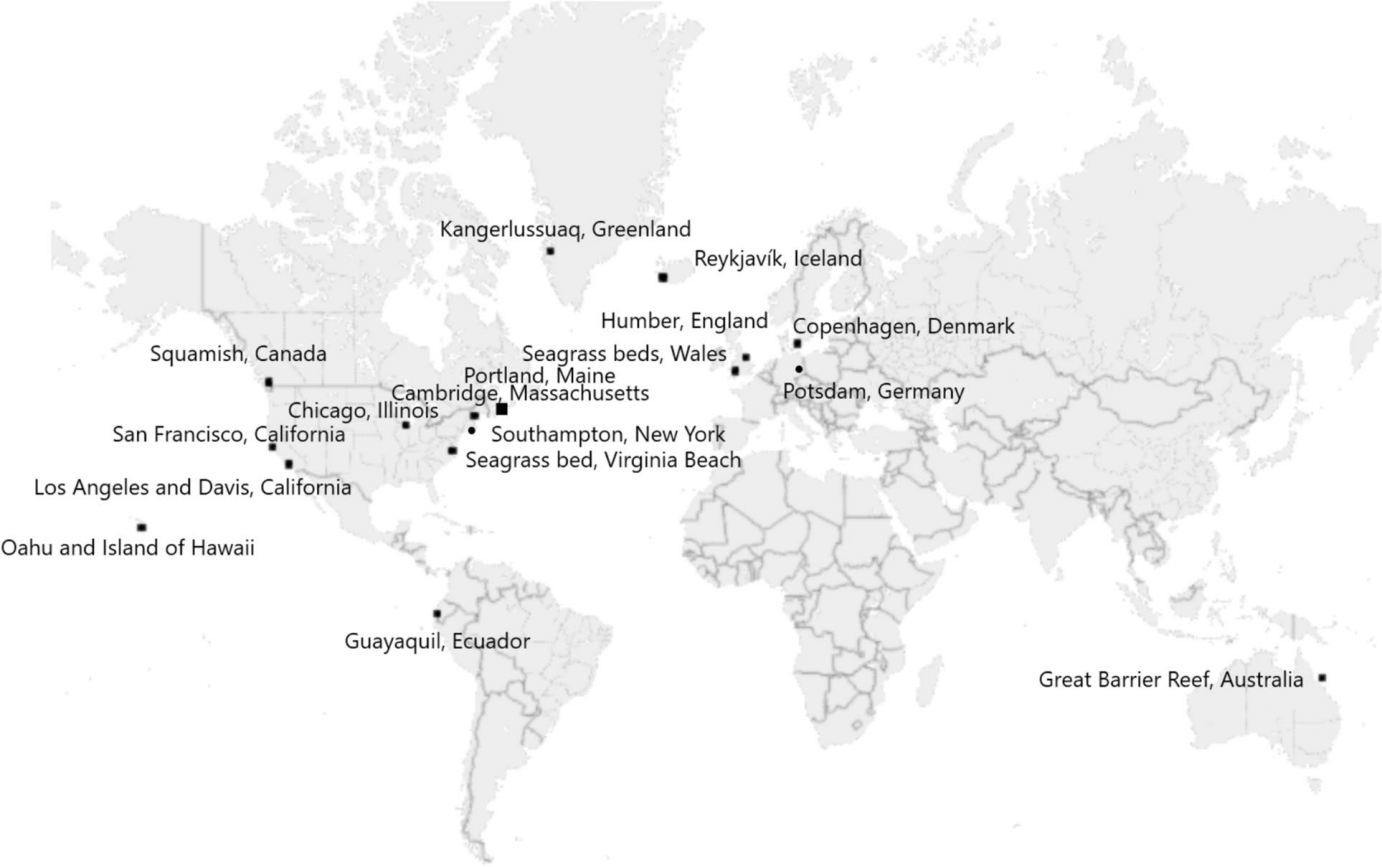
Global location of twenty carbon removal and solar radiation modification experiments. Source: Authors

We selected 20 distinct cases of carbon removal and/or solar radiation management activities (see Table 1 and Fig. 1). Cases reflect a mix of technological, geographic, and sectoral diversity along with familiarity by the authors and availability of data, including the opportunity to conduct a site visit and onsite interviews. Although including such a range of both carbon removal and solar radiation management interventions could be subject to contestation, we believe in examining all 20 cases available as part of a portfolio for six reasons. Firstly, both options can work together as part of an effective strategy for storing emissions (carbon removal) and lowering temperature (solar radiation modification). Secondly, the pathways can directly affect each other, with one recent study noting that solar radiation modification techniques can enhance the carbon storage potential of various forms of CDR (Zhao et al. 2024); conversely, solar radiation modification techniques such as aerosol injection can affect plant growth and water availability which in turn affect nature-based sources of carbon removal (SAPEA 2024). Thirdly, both options reflect the actual dilemma policymakers face in choosing which technical options they want to pursue to address climate change with limited resources and great uncertainty. Fourthly, they “match” the scope and scale of the problem of climate change. Fifthly, many of our 20 projects included multiple or both types of interventions. Sixthly, we worry that excluding one set of options (such as solar radiation management) from the discussion artificially narrows and censors debate, which could have a result of pushing deployment to only the least responsible actors and locations (Sovacool et al. 2024a).

**Table 1:** Overview of twenty carbon removal and solar radiation modification experiments

No.	Date	Climate intervention(s)	Project location visited	Reference name used in this study	Expert interviews conducted (*N* = 118)
1	July 2021	Ice protection	Greenland Ice Sheet, Kangerlussuaq, Greenland	Arctic ice protection	4
2	September 2021	Direct air capture	Climeworks Orca Direct Air Capture facility, Hellisheiði, Iceland	Orca DAC	3
3	April 2022	Bioenergy with carbon capture and storage (BECCS)	Drax BECCS facility, Humber, England	Drax BECCS	10
4	June 2022	Afforestation, community forestry, and mangrove restoration	Various forests near Guayaquil, Ecuador	Ecuadorean forestry	5
5	October 2022	Direct air capture	Carbon Engineering Direct Air Capture facility, Squamish, Canada	Carbon Engineering DAC	3
6	October 2022	Marine cloud brightening, coral reef fogging and shading, and ecosystem adaptation and restoration	Great Barrier Reef, Northern Queensland, Australia	Reef Restoration and Adaptation Program	23
7	February 2023	Stratospheric aerosol injection	SCoPEx Laboratory, Harvard University, Cambridge, Massachusetts, USA	Harvard SAI	4
8	March 2023	Seagrass restoration and marine carbon removal	Various sites across Project Seagrass, Wales, UK	Project Seagrass	12
9	March 2023	Seaweed planting, kelp cultivation, carbon buoys, ocean alkalinization, coastal protection, and beach nourishment	Running Tide, Portland, Maine, USA	Running Tide	11
10	July 2023	Enhanced rock weathering, glacial rock flour	Centre for Rock Flour Research, Taastrup and Copenhagen, Denmark	Rock Flour Weathering	2
11	August 2023	Carbon removal for cement and concrete	CarbonCure Carbon Removal at the Ozinga Cement Facility, Chicago, USA	CarbonCure	10
12	October 2023	Ocean alkalinity enhancement, coastal carbon capture, beach nourishment, and coastal protection	Vesta (formerly Project Vesta), Southampton, New York	Vesta	5
13	November 2023	Seagrass restoration and blue carbon, coastal adaptation and protection in the Chesapeake Bay and Eastern Shore	Brownsville and South Bay, Virginia, USA	Virginian Seagrass	9
14	November 2023	Ocean alkalinity enhancement	University of Honolulu and Hawaii Institute of Marine Biology in Kaneohe, Hawaii, USA	Hawaiian OAE	4
15	November 2023	Reforestation and restoration of native forests	Terraformation, Kailua-Kona and Kohala, Hawaii, USA	Terraformation	4
16	November 2023	Bio-oil sequestration, carbon capture and storage, BiCRS (biomass carbon removal and storage)	Charm Industrial, San Francisco, California, USA	Charm Industrial	2
17	November 2023	Enhanced rock weathering	Working Lands Innovation Center (WLIC), University of California Davis, California, USA	WLIC	1
18	November 2023	Direct Ocean Capture	Captura, Port of Los Angeles, California, USA	Captura	3
19	November 2023	Direct Ocean Capture using seawater electrolysis	Equatic (formerly SeaChange), Port of Los Angeles, California, USA	Equatic	1
20	January 2024	Biochar, carbon–neutral cement	BNB, Potsdam, Germany	BNB Biochar	4

Source: Authors

The authors then conducted original site visits, formal and informal expert interviews, and naturalistic observation at each of the 20 case examples. For each, we followed an interview script focused on the seven following questions:

1. Technical performance: How does the technology being site visited work?
2. Innovation and learning: What patterns and forms of experimentation have shaped the technology? What objectives and aims are most central for ongoing work here?
3. Barriers: What barriers exist, including those related to scaling and commercialization? How well are the risks and side effects understood?
4. Policy and governance: Have any lessons/policy recommendations emerged so far? What are they?
5. Future deployment: Realistically, how much of an impact can this technology make on addressing climate change (whether for temperature targets or emissions reductions)? By 2030? By 2050?
6. Public and stakeholder involvement: To what extent are the public and other stakeholders involved in/consulted on the research, development, and deployment of this technology? If so, how? What efforts and/or initiatives are being made here?
7. Messaging/strategy: If you wanted the public/policymakers to know one thing about this technology, what would it be?

Informal expert interviews ranged from 10 to 20 min and formal expert interviews ranged from 25 to 180 min, with some being more conversational and shorter in duration while the site visit was ongoing. This technique of convivial interviewing is commonly used in the fields of participatory action research and ethnography, and it can enable a more flexible and dynamic interviewing protocol which can minimize bias (by making the interview more accessible to respondents) and reduce the transaction costs involved with undergoing an interview (Burgess-Limerick and Burgess-Limerick 1998; Kindon et al. 2007; MacDonald 2012; van Enk 2009; Martiskainen et al. 2020). We call them “expert” interviews as our sampling strategy targeted those centrally involved with each of the 20 case study projects—representing positions in research, technology development, and (project, institute, or firm) leadership. We did not sample respondents from the public at large. Experts spoke to themes such as social license to operate, the involvement of the public, and degrees of social feasibility or acceptance for their cases, but in those instances, they were speaking on behalf of the public.

**Fig. 2:**
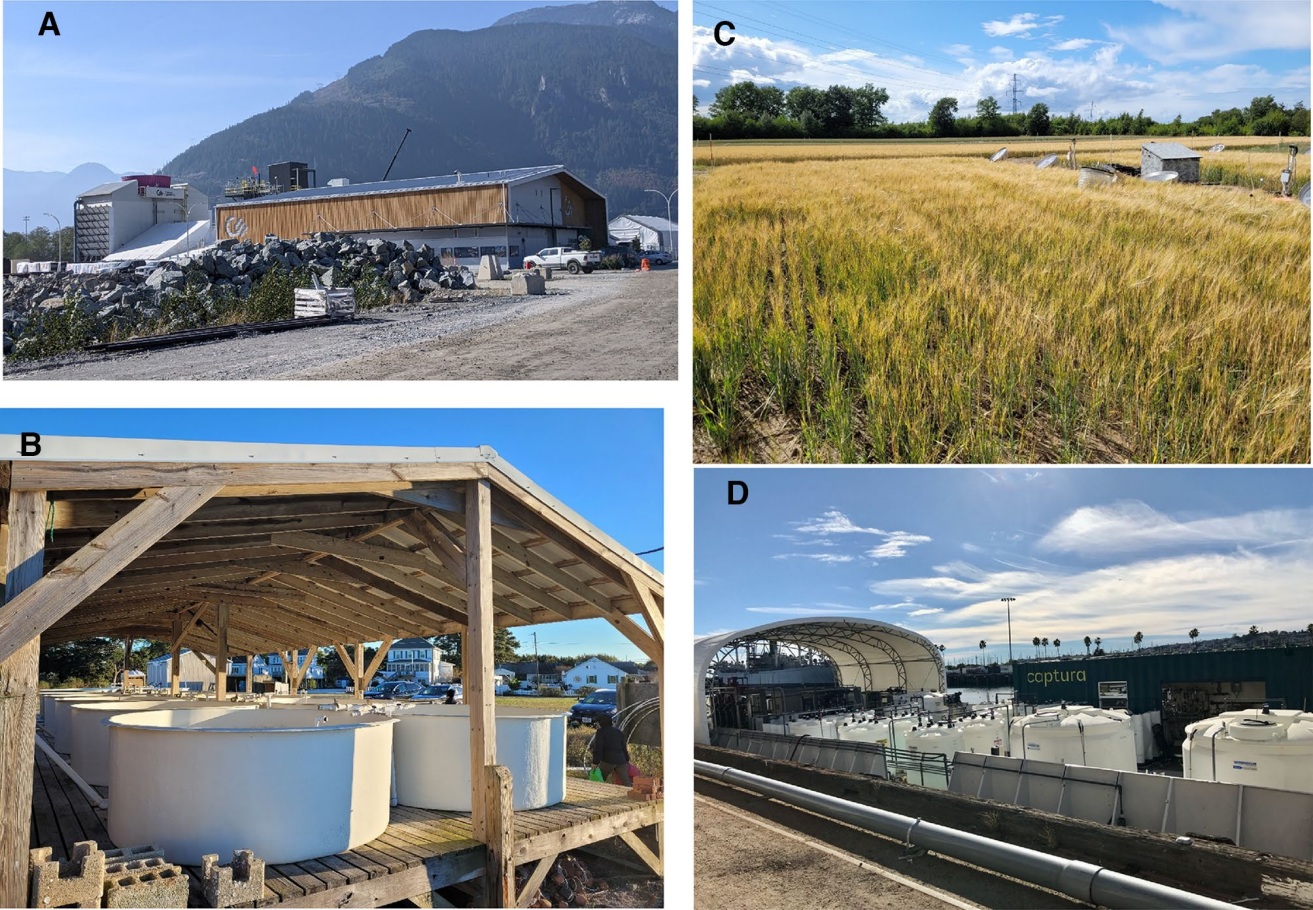

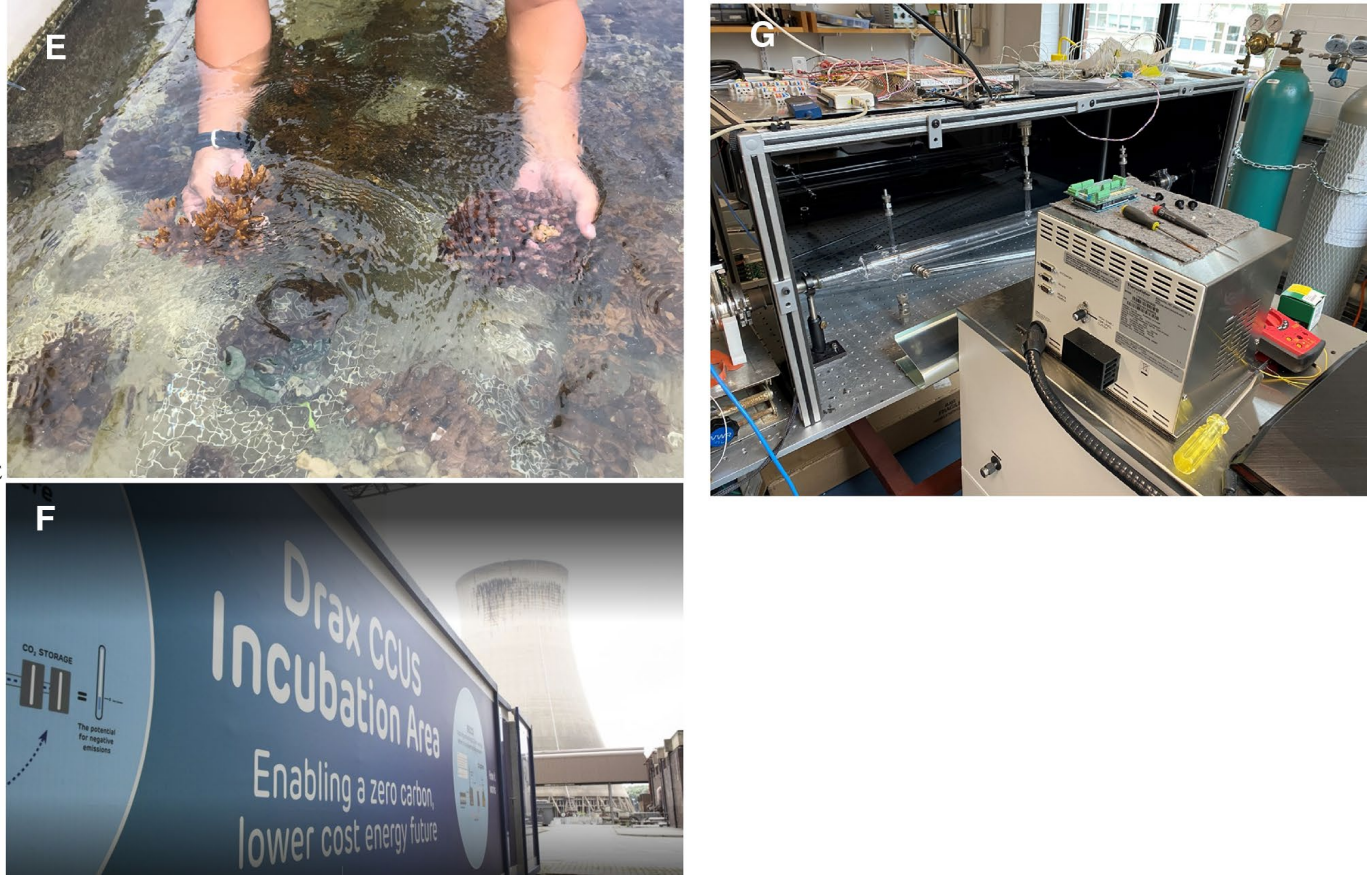
A sample of climate intervention experiments where naturalistic observation was undertaken. Source: Authors. *A* the Direct Air Capture facility in Squamish, Canada, *B* seagrass restoration ongoing of the coast of Virginia. *C* glacial rock flour experiments in Denmark, *D* Captura in Los Angeles, *E* ocean alkalinity enhancement in Hawaii, *F* the Drax BECCS facility in the UK, *G* a stratospheric aerosol injection simulator at Harvard University

Interviews were triangulated with naturalistic observation: a form of revealed preferences analysis (people are observed indirectly) distinct from stated preference analysis (where respondents are asked questions directly) that is commonly used in ethnography and anthropology (See Fig. 2). Observation is one of the foundational modes of qualitative research inquiry (Angrosino 2007). Unlike interviews, which solicit stated preferences in a contrived setting, naturalistic observation permits the assessment of revealed preferences in a realistic and lifelike setting (Carey et al. 2020).

In collecting our qualitative data, we adhered to ethical protocols throughout our site visits, observations, and interviews. Interview participants knew in advance they were being observed and were given information on the project prior to meeting. Moreover, all participants gave either written or verbal consent (often recorded with a digital audio recorder) to proceed with the interview, and to confirm they gave permission to be recorded. To protect participants and to ensure candor during the interviews, all information was treated as confidential and is presented anonymously throughout the paper. Any photographs utilized also have been edited or cropped to avoid revealing any identifying information about participants. Lastly, given this research was hosted at a European university, all data has been processed and handled according to the requirements of the General Data Protection Regulation.

### Analytical rubric and thematic analysis

With our interview and site visit data collected, we created an analytical rubric with several dimensions: (a) actor and stakeholder types, (b) the promises inherent in the interventions, (c) innovation dynamics, and (d) co-impacts.

Actor coalitions refer to groups of people or organizations that share similar beliefs and work together to influence social, political, or technical change (Pierce and Osei-Kojo 2022; Weible et al. 2020). To identify them, we employ a typology of stakeholders, building on Gamble et al. (2021) and Bukirwa et al. (2024), which consists of those deploying the technology, those doing policy, planning, and permissions, or implementing and monitoring, as well as those doing outreach and engagement. This resulted in a list of 24 specific stakeholder groups summarized in Table 2.

**Table 2:** Typology of five stakeholder types and 24 specific stakeholder groups utilized to identify actor coalitions

Regulation and government	Local government
	National government
	Intergovernmental bodies
Funders and corporate firms	Banks
	Institutions giving prizes and awards
	Local businesses
	For-profit companies
	Energy suppliers
	Technology providers
Knowledge production	Museums
	Research institutes
	Patent offices
	Universities
	Schools and lower educational colleges
Civil society	Community groups
	Non-governmental organizations (NGOs)
	Environmental justice groups
	Labor and unions
Publics	Direct users of the technology (technology specific)
	Local residents (place specific)
	General public (across entire country or region)
	Artists
	Youth
	Farmers and/or traditional landowners

Source: Modified from Gamble et al. (2021) and Bukirwa et al. (2024)

To explore the promises and requirements associated with our 20 cases, we engage with concepts arising out of the *sociology of expectations*. This approach aims to assess how “guiding visions” or “normative expectations” about future benefits affect and structure technology (Van Lente 1993; Bakker et al. 2011). Expectations can be individual or collective, and they reveal the “narrative infrastructure” or “mosaic of stories” surrounding technologies (Deuten and Rip 2000). To build support for new technologies, actors often make promises about what the technology can do, to attract funding but also form a vision about that technology’s potential. These promises can take many forms: umbrella promises tend to be vague, open ended, and non-falsifiable; more specific promises can be closed and falsifiable, and thus more prone to disappointment. Such promises culminate in a *promise–requirement cycle*. Promises become part of an agenda-setting process that germinates into a requirement for engineers and other actors, giving them a “mandate” to develop “their” technology (Bakker et al. 2011). In this way, promises about the technology become twinned to addressing certain problems which then in turn create pressure for developers and advocates to deliver on that technology.

To explore the *innovation dynamics and styles* of the 20 cases, we draw upon insights from the innovation studies, technological diffusion, and clean-technology development literatures (Sovacool et al. 2023c;  2024b). Though vast, this literature discusses the salience of at least eight dimensions to innovation. *Coupling* refers to the integration of approaches across different sectors and direct and ancillary techniques involved (Baum et al. 2023). *Expertise* involves the degree of specialized knowledge needed to research, test, and (potentially) deploy each intervention (Mahony and Hulme 2018; Maliniak et al. 2021). *Cost and capital intensity* reflect how expensive the intervention is, as well as whether it is rather technology intensive (direct air capture) or labor intensive (seagrass planting) and its degree of modularity and granularity (Wilson et al. 2020; Giannousakis et al. 2021). *Temporality* refers to whether it can be deployed and/or capture emissions or achieve reductions in temperature in the near term, mid-term, or long term (Buck et al. 2023; Burke and Gambhir 2022), as well as how permanent its efficacy is, or if it has the risks of termination shock or reversibility (Chiquier et al. 2022; Sovacool et al. 2022; Ruseva et al. 2020; Herzog et al. 2003). *Location* refers to whether the current experiments are outdoor vs. indoor, or are undertaken only in model simulations (Low et al. 2022). *Scaling* involves the techniques being considered for scaling up or commercial deployment (Nawaz et al. 2024; Reynolds 2019; Field and Mach 2017; Irvine et al. 2016). *Engagement* refers to the extent that local communities, laypersons, citizens, and/or specific stakeholders are involved in the experiment, as well as whether it has perceived legitimacy and a social license to operate among the general public (Low et al. 2024; Fritz et al. 2024; Hilser et al. 2024; Cooley et al. 2023; McLaren and Corry 2021; Frumhoff and Stephens 2018; Peters et al. 2012). Rather than being fixed or dualistic, all these innovation attributes can exist on a spectrum across open (bottom-up, grassroots, and inclusive) versus closed (top-down, corporate, and proprietary) (Christiansen et al. 2023; Battersby et al. 2022).

Lastly, to explore the perceived and prospective *co-impacts*, we draw upon the climate science literature (Babiker et al. 2022; Ürge-Vorsatz et al. 2014) as well as new studies specifically on the co-impacts of climate intervention (Sovacool et al. 2023b). For symmetry, this literature examines both desirable and undesirable outcomes and aspects for each climate intervention. This literature broadly suggests that the costs and benefits of climate interventions can involve *financial and economic* co-impacts such as the expansion of markets, business models, government revenues, and phantom carbon credits (among others); *socioenvironmental* co-impacts such as protection of habitats, forests, oceans, or species, or the provision of decent work and high paying jobs or generate negative implications for biodiversity or land use conflicts; *technical* co-impacts such as the improved performance of systems, disruptive or positive innovation patterns for a sector, enhanced efficiency, or positive and negative learning and experimentation; or *political and institutional* co-impacts such as the achievement of policy goals (relating to industrial strategy, energy security, equity, and “leveling up”, a term used to describe processes that reduce the economic imbalances between areas and social groups), expanded sovereignty, or the creation of a moral hazard. In total, the assessment along these multiple dimensions enables us to comprehensively map and examine the emergent space of selected climate intervention experiments and activities.

### Limitations

Our research design does have methodological and conceptual limitations. Methodologically, we limited our interview data collection to experts, meaning we did not include members of the public or affected communities directly. We did not seek a representative sample of respondents, given constrained resources in terms of time and person power, but also given the large number of sites visited, which would have made seeking a representative sample of respondents for all cases prohibitive. Instead, we sought a purposive sample based primarily on those with expert knowledge of a given pilot or project. Thus, we cannot claim that our results have reached saturation across all cases and, moreover, in some cases, the number of respondents is much smaller, e.g., in the single digits, compared to others. We have tried to offset this limitation by triangulating the purposive sample of interview data with the site visits, naturalistic observation, and wherever possible document analysis of project related articles or reports, which we read to orient ourselves to each case study along with our interview data and site visit observations.

In this paper, we maintain a conceptual focus on sociotechnical promises, innovation dynamics, and perceived co-impacts. However, we recognize that there are dozens of sociotechnical theories and heuristics beyond these that future researchers could use. Sovacool and Hess (2017) surveyed sociotechnical theorists themselves and identified 96 relevant theories; Sovacool et al. (2023b) examined a narrower scope of net-zero and industrial decarbonization theories through an expert-guided review and identified 88 relevant theories. These include theories of path dependence and lock-in (Morrison et al. 2024), the social construction of technology (Sovacool et al. 2023d), polycentric governance (Oberlack et al. 2018), and others.

## Results and discussion

This section presents our thematic results from the site visits and interviews described in Sect. "Case study selection and data collection" with the analytical rubric and themes summarized in Sect. "Analytical rubric and thematic analysis".

### Actors, stakeholders, and actor coalitions

Collectively, as Table 3 summarizes, 133 distinct stakeholders were identified by our interviewee respondents as relevant to the 20 climate interventions. Across all 20 cases, the stakeholders most identified as being relevant were local government (for half of the cases), national government (half), and for-profit companies and firms (half), as well as universities (half). This was followed by local businesses (8 of the 20 cases), community groups (7), and farmers and landowners (7). The two projects where the most stakeholders came up during the interviews were the Reef Restoration and Adaptation Program and Project Seagrass, each had 16.

**Table 3:** Stakeholder analysis for 20 climate and climate intervention cases

	Local government	National government	Intergovernmental bodies	Banks	Institutions giving Prizes	Local businesses	For-profit companies	Energy suppliers	Technology providers	Museums	Research institutes	Patent offices	Universities	Schools and colleges	Community groups	NGOs	Environmental justice groups	Labor and unions	Direct Users	Local residents	General public	Artists	Youth	Farmers and traditional landowners
Arctic ice protection							X												X					
Orca DAC	X						X	X	X				X											
Drax BECCS	X	X				X	X	X										X		X				X
Ecuadorean forestry	X	X													X	X	X	X						X
Carbon Engineering DAC							X	X	X			X												
Reef Restoration and Adaptation Program	X	X	X			X				X	X		X	X	X	X	X			X	X		X	
Harvard SAI					X				X		X	X	X			X								
Project Seagrass	X	X	X			X				X	X		X	X	X	X	X		X	X	X	X	X	
Running Tide				X	X							X												
Rock Flour Research		X					X				X		X		X			X	X		X			X
CarbonCure						X	X		X				X											
Vesta	X	X				X	X		X				X	X	X	X			X	X				
Virginian Seagrass	X	X				X					X		X	X	X	X				X			X	X
Hawaiian OAE	x	X											X											
Terraformation										X				X	X					X	X			X
Charm Industrial	X	X				X									X			X						X
WLIC	X										X				X				X					X
Captura	X	X		X			X		X		X	X		X										
Equatic	X	X				X	X		X															
BNB Biochar						X	X	X	X			X	X											

Source: Authors, based on interview data (*N* = 118 respondents) described in Sect. “Research design and analytical rubric” as well as observations during site visits and the expert knowledge of the author team concerning project literature, when relevant. An X indicates that the stakeholder was deemed relevant or important to a particular project. When comparing across cases, differences in the number of interviews need to be considered

Interestingly, these disparate actor types often form into identifiable actor coalitions. Virginian Seagrass sees a strong involvement of regulatory and government stakeholders, given the set requirements for policy, planning, and permissions for seagrass restoration: the Virginia Department of Environmental Quality (DEQ), Virginia Marine Resources Commission (permitting, public reviews), local governments and municipalities (e.g., Virginia Beach, in their role as lease holders of “bottoms”), federal agencies (Army Corps of Engineers). As one interview respondent explained, “the bottom in Virginia, everything below mean low tide is owned by the state, so the state of Virginia actually owns any carbon credits out here.” Other experiments, such as Orca DAC and Carbon Engineering DAC, have a preponderance of funders and corporate firms noted as stakeholders, given that they seek to license their technology to a range of industry actors.

Charm Industrial (BiCRS), by contrast, possesses an actor coalition involving local businesses, mostly farmers and forest managers providing biomass. As one interview respondent explained, “we need some sort of supplier on biomass. We may need different contractors based on what and where we’re operating. For example we may need a trucking organization to help us with the transportation of the bio-oil, but for the most part we are vertically integrated from first acquiring the biomass to turning it into bio-oil to doing the injections, almost all of that is managed by Charm.” Terraformation (reforestation) sees a very visible role for civil society stakeholders from the restoration sector such as the Milwaukee Bishop Museum and Ecological Restoration Camp. As part of Australian Reef Restoration and Adaptation Program, the actor coalition includes Traditional Owners as well as tourists, youth, and members of the public, notably in uploading data on reef stress and mortality and in supporting citizen science efforts. Vesta also reported an actor coalition including the North Sea Beach Colony, where activities took place, coastal landowners and communities including an annual meeting with 50–60 houses, a Peconic Estuary Partnership, and an internship program with local high school students on Long Island.

Some, like Equatic (Direct Ocean Capture), did not strongly emphasize stakeholder engagement or the strength of their actor coalitions, with one interview respondent admitting that “public engagement has not been a focus so far.” The Hawaiian research project on ocean alkalinity enhancement was similarly limited to a narrower actor coalition of university researchers and their experiments on how olivine (magnesium-based rock), limestone (calcium-based rock), and sodium hydroxide (strong base) impact corals. This might reflect the rather early stages of research underway, as the need for engagement of local, Indigenous communities, and public authorities, albeit at later stages, was acknowledged.

In terms of scale, some experiments were notably trans-local and multi-scalar. Harvard’s SAI experiments involve a global advisory board—perhaps not surprising given the backlash such activities had aroused at an earlier stage (Oksanen 2023). Running Tide (macroalgae cultivation for deep ocean storage) has research and deployment activities in the Midwestern USA, off the coast of Maine, in Iceland, and in the high seas of the deep ocean. In enhanced weathering, the Center for Glacial Rock Flour Research involves the Globe Institute, led by Minik Rosing, the Rock Flour Company (a start-up, spinoff from academic research), the Greenlandic government involved around extracting and mining, and small holder farmers (in Ghana, five growing seasons, maize), collaborating researchers, and working farms for trials in Denmark (one completed: maize, potatoes, spring wheat; two more starting in 2024: first maize) and Australia (maize, sugarcane, barley, legume), along with Carlsberg A/S (collaborator on earlier Danish barley trials), and a testing laboratory for grinding machinery (in Austria).

### Promises and requirements

The inherent promises and requirements of climate intervention technologies were connected intimately to a multitude of dimensions and problems shown in Table 4. Every single experiment was connected to addressing numerous interrelated problems—with climate change often only being one example.

**Table 4:** The sociotechnical problems and promises inherent in 20 climate intervention experiments

Dimension	Arctic ice protection	Orca DAC	Drax BECCS	Ecuadorean forestry	Carbon engineering DAC	Reef restoration adaptation program	Harvard SAI	Project seagrass	Running tide	Rock flour research
Technological and scientific	To facilitate better Arctic climate modeling capabilities	Knowledge of materials and high- vs. low-temperature DAC processes	Advancing knowledge of biomass gasification	Improving estimations for carbon storage	Knowledge of materials and high- vs. low-temperature DAC processes	Realtime data on reef health, weather forecasting	Knowledge of aerosol mixing in the stratosphere	Enhancing basic science for seagrass breeding and resilience	Advancing science about mycelium, macroalgae genetics, and ocean currents	Better understanding of soil system dynamics
Socioeconomic	Enhanced tourism revenues	Capturing the nascently emerging DAC market	Provision of domestic energy sector jobs	Community co-benefits including employment and economic diversification and resilience	–	Enhanced tourism revenues, provision of jobs	–	Improved coastal resilience, fisheries productivity	Capturing monetary incentives from possible blue carbon credits	Economic growth and job opportunities for Greenland, working farms
Environmental	Addressing sea level rise, protecting glaciers and ice sheets	Reversing climate change	Achieving climate change mitigation goals, meeting domestic carbon budget	Better land-use management, including soils and water	Reversing climate change, selling sustainable aviation fuel	Strengthening reef resilience, enhancing the carbon storage potential of marine habitats	Lowering temperature stress, heat extremes	Enhanced carbon storage, water filtration, fisheries stability and reduced ocean acidification	Reversing climate change, reducing ocean acidification, improving coastal resilience	Concerns over fertilizer use, toxicity, and human health hazards
Security	Minimizing tundra loss and potentially protecting Arctic military bases and experiments	–	–	Lessening the risk of illegal and criminal forest activities	–	Avoiding the complete collapse of local economies, and illegal fishing and poaching on the Great Barrier Reef	Enhancement of aerospace sector and possible defense applications	–	Enhancing fleet deployment capabilities for the US Navy, improvements in low-orbit satellites	Improvements to vulnerability for smallholder farms
Political	–	–	Helping the Humber region “Level Up” goals and meeting industrial strategy and local environmental goals	Reduced unemployment in rural areas and thus the risk of populism, strikes, and disruption	–	Preserving and maintaining a national and global heritage site	–	Improved resilience of rural areas and meeting some of the “Leveling Up” goals of Wales	–	Opportunities away from Danish “colonialism”

Dimension	CarbonCure	Vesta	Virginian seagrass	Hawaiian OAE	Terraformation	Charm industrial	WLIC	Captura	Equatic	BNB biochar
Technological and scientific	–	Technical understanding of carbon sequestration	Understanding and mapping of seagrass health, breeding of more resilient varieties	Knowledge about marine ecosystems and amounts of carbon sequestration	Knowledge of native and biodiverse seeds	Learning from continuous experimentation and improvements	Knowledge about soil health and sequestration potential	Understanding the capacity of the ocean for carbon storage	Advancements in seawater electrolysis and hydrogen production	Biochar storage efficacy and “bang for buck”
Socioeconomic	Maintenance of jobs in the cement and concrete sector	–	Job creation/avoiding job losses + opportunities for local communities	Could capture monetary incentives from possible blue carbon credits	Ability to generate large amounts of carbon credits	Transferring skills and workforce from the fossil industry, and job creation in regions in transition to fossil phase-out	Reduced costs for farmers and opening up of new revenue streams	Capturing monetary incentives from possible carbon credits	Capturing monetary incentives from possible carbon credits	Capturing a larger percentage of the low-carbon buildings and materials markets
Environmental	Long-term and durable carbon storage in building materials	Coastal erosion and environmental sustainability	Need for climate resilience and adaptation	Climate change, ocean acidification	Biodiversity, prevention of soil erosion	Better land use management practices, reduced risk of wildfires, prevention of methane leaks by closing old oil wells	Carbon storage can be combined with soil amendments, can enhance agricultural productivity and reduce fertilizer and water use	Potentially reducing ocean acidification as the water discharged is of slightly increased alkalinity	No environmental harm as water discharged into the ocean is of similar composition to the sea water used	Reducing waste, substituting for carbon-intensive materials in roads and buildings
Security	–	–	–	Internationally agreed upon rules are needed to avoid a race between actors operating under different jurisdictions	–	Reducing the hazards of future forest fires	–	–	–	–
Political	–	–	–	Need for international regulatory frameworks	Better regulation to avoid reproducing the mistakes made on markets for offsets	Long-term political commitment and need for technology agnostic/open policies that incentivize development and deployment of CDR	Change in agricultural policies and land ownership, and incentives for framers to adopt	Long-term political commitment, moving beyond voluntary markets, and need for technology agnostic/open policies that incentivize development and deployment of CDR	Long-term political commitment and need for technology agnostic/open policies that incentivize development and deployment of CDR	–

Source: Authors, based on interview data (*N* = 118 respondents) described in Sect. “Research Design and Analytical Rubric”. A dash “–” indicates that the particular problem or promise did not arise from our interview material, even if it may still be relevant or important to a particular project

Some promises fall into the technical and scientific dimension, and relate to better modeling capabilities, forecasting techniques, first-order learning, or the filling of knowledge gaps, or, simply the ease with which experiments could achieve scaling up. As one respondent spoke of Terraformation: “Reforestation, restoration of native forests at large scale, that is the simplest and most realistic contribution to tackling climate change.” A respondent for Captura (Direct Ocean Capture) stated that their approach is “harnessing the carbon drawdown mechanism of oceans to remove excess atmospheric CO2 without any by-products or ocean additives as inputs”.

Other requirements and promises fall into the socioeconomic domain, involving aspects such as capturing new markets, enhancing tourism (Reef Restoration and Adaptation Program, Virginian Seagrass, Arctic ice protection), providing jobs or avoiding job losses (Virginian Seagrass, CarbonCure), and reducing the costs of deployment (Carbon Engineering, Climeworks; both DAC). One respondent involved with the Reef Restoration and Adaptation Program work stressed that there are proposals for marine cloud brightening where “you target it for tourist hotspots”, both because of the economic importance, locally and nationally, of these areas and the opportunities for deployment: “you’ve got all those tourist boats that deploy every time we’re out there to meet you.” Similarly, for Virginian Seagrass, positive outcomes of seagrass restoration centered on the growing number of tourists “who would come here to go birding” or “rent a boat or go on a scallop tour”. At the same time, this can cut both ways, as noted for the Reef Restoration and Adaptation Program, where salient concerns about climate intervention included that “it’ll stop tourism, it’ll ruin jobs, people will stop investing in the reef with the environmental funds”.

Although addressing climate change emerged as a paramount environmental consideration of the assorted activities, other environmental issues were also framed as being addressable, including sea level rise (Virginian Seagrass, Project Seagrass, Reef Restoration and Adaptation Program, Arctic ice protection), deforestation (Ecuadorian afforestation), enhancing coastal resilience (Virginian Seagrass, Project Seagrass), reducing ocean acidification (Vesta, Rock Flour Research; (marine) enhanced weathering), lowering wildfire risk (Charm Industrial, BiCRS, bio-oil sequestration), and improving biodiversity protection (Reef Restoration and Adaptation Program, Virginian Seagrass). For example, one respondent articulated that the primary benefit of Virginian Seagrass restoration was “a caretaker pathway towards whole system restoration and climate resilience for the entire coast.”

Envisioned political dimensions were expansive. Promises related to security included protecting military bases or enhancing naval fleet deployment (Running Tide, macroalgae), or enhancing the capacity of aerospace firms (Harvard SAI, solar geoengineering). Political promises involved poverty reduction (Drax BECCS), enhancing national competitiveness and industrial strategy goals (Drax BECCS), minimizing populism, or reversing historical colonialism (Rock Flour Research).

### Innovation dynamics and styles

The first innovation dimension examined was *coupling*—and experiments revealed a startling array of connections to different sectors and technologies (Baum et al. 2023). Respondents spoke about how both Orca DAC and Carbon Engineering DAC (recently bought by Occidental Petroleum) have strong couplings to geological storage and enhanced oil recovery as well as (for Carbon Engineering) aviation and maritime fuels. BNB biochar is coupled with industrial wax, carpentry, and 3D printing as well as waste remediation and building materials such as concrete and cement, a coupling also evident in CarbonCure. Project Seagrass, Virginia Seagrass restoration, and Reef Restoration and Adaptation Program are all coupled with recreation and tourism as well as fisheries and the maritime sectors; the Reef Restoration and Adaptation Program and Running Tide are also coupled to the promise of coral reef restoration and Hawaiian research ocean alkalinity enhancement’s aims at understanding its effect on corals and potentials for restoration. Both enhanced rock weathering projects have strong couplings to mining (as an input) and soil management and improved farming and agricultural productivity (as an output). Equatic (Direct Ocean Capture) is strongly coupled to hydrogen manufacturing; Captura (Direct Ocean Capture) and Terraformation (reforestation) in different ways to water desalinization, in the case of Terraformation watering plants in water-scarce regions. Some experimental innovations are even coupled to each other: with seagrass restoration linked to creating biochar (United Nations Environment Program 2020), and biochar as well as macroalgae coupled to enhanced waste capture for coal and enhanced production of bioenergy (Cole et al. 2014). Bio-oil production of Charm Industrial (BiCRS) is also coupled with biochar.

The second dimension was *expertise* required for each experiment. Atmospheric science and aerospace engineering dominate for Harvard SAI, for instance, whereas marine science and biology dominate for seagrass and reef restoration. Both DAC projects remain engineering focused, with efforts focused on standardized design but also highly specialized equipment (e.g., Visionary Vendors ™ provides 6 types of equipment to Carbon Engineering DAC). BNB Biochar is more oriented toward materials science, along with focusing on potential issues with waste source, while for enhanced rock weathering next to soil sciences also experience-based knowledge of farmers and indigenous groups is highlighted.

In terms of the underlying CDR approaches, enhanced rock weathering, biochar and even afforestation are connected to soil sciences, biology and ecology. Seagrass restoration in both Virginia and Wales by contrast is much lower tech in terms of distribution (people can do it by hand), although research efforts are increasing, to diverging extents across actors and projects, related to improving effectiveness of seed harvesting/curing and processing/ storage/planting: these involve, e.g., understanding seed quality and system dynamics such as bathymetry as well as improving seed quality (enhanced brood stocks, cross-breeding, more stress-tolerant varieties) and more concern for climate conditions, e.g., whether to seed in fall or spring as well as whether to use “freshwater shock” or let seeds germinate as normal. Vesta ocean alkalinity enhancement), in line with their goal of attaining carbon credits, demands a reasonably high-level of expertise to demonstrate that carbon is being stored (along with understanding dynamics of sand movement and assessing influence on sea life): e.g., extensive laboratory analysis including olivine dissolution, X-ray fluorescence, pH and alkalinity testing, and ecological testing of impacts on horseshoe crabs, oysters, and potential for toxicity. Charm Industrial (BiCRS) requires specialized knowledge of how fast pyrolysis works (how to operate and maintain their machine); in the case of woody biomass, knowledge of how to sustainably manage forests and prevent wildfire; and specialized knowledge needed for geological storage of the bio-oil, where they build on expertise from oil and gas.

Equatic (Direct Ocean Capture) requires knowledge of engineering but also chemistry, hydrology and water science, as they seek to build on water filtration techniques. The Reef Restoration and Adaptation Program, Running Tide (macroalgae) and Hawaiian ocean alkalinity enhancement all involve marine biology, oceanography, ocean chemistry, and (for Running Tide) mycology and wave science. Looking horizontally across all 20 projects, two recurring epistemic dilemmas arise: generalizability and accuracy. To the first, respondents did question that even if “one experiment is demonstrated to work at one time in one area, one cannot assume that it will necessarily work in all areas equally.” To the second, there is a mismatch between the nature of current experiments and what they are trying to simulate; Harvard SAI researchers are trying to simulate what storms and volcanic eruptions do, but there is, as one respondent put it, “arrogance and hubris in thinking the entire stratosphere can be represented in a small one-meter glass tube in a lab.” Such circumspection is particularly notable given how it tends to contradict and run against the efforts in many projects to underscore the standardization and calculability of their approaches, i.e., to be awarded carbon credits.

A third dimension is *cost and capital intensity*. Some experiments, such as the DAC initiatives, BNB Biochar, or Harvard SAI, are technology intensive or capital intensive. As one biochar respondent put it, “Initial investment is quite high, but otherwise not so many risks.” One of our Harvard SAI respondents added that “the perception is that SAI is cheap and fast, but in reality the full cost is greater than it seems, as deployment would require a whole new observation network, satellites, modeling, that tries to track injections, a million tons of SO2 [sulfur dioxide], how effective radiative forcing is –reality is more expensive than you think, [there is] hardly a satellite you can launch for less than $50 million [and] net cost could be magnitudes of order higher; Planes and delivery are not the problem, this net cost and infrastructure is a severe limitation.”

Others such as seagrass restoration or reef restoration are relatively labor intensive, as one Project Seagrass respondent noted, “a seagrass seed costs less than a cent and can be literally distributed by hand.” These add up, however, with one Virginia Seagrass respondent noting that collectively “We planted eighty acres of 8 million seeds in two days working out of two boats for not terribly long days, like, not huge crews, it’s pretty easy doing it how we do it.” Glacial rock flour is similarly labor intensive, at least using the procedures established for the trials, as it is already “pre-ground” to 2.6 microns, before being spread by laborers (i.e., researchers) manually across fields—this is broadly like the approach of WILC. Terraformation (reforestation) is also labor intensive in terms of the collecting of seeds for the seed bank and processing them; also, the plant nursery is labor intensive, as is the actual reforestation.

Some, like Vesta (marine-based enhanced weathering), are intermediate between the two: at the research or field trial stage, it is very labor intensive with most of the application being done manually (by Vesta employees, using front loaders) along with daily collection of sediment cores and registering sensor measurements. Looking to the future and aims of scaling-up, however, key costs envisioned include sourcing and grinding of rocks—of note, the state of glacial rock flour avoids such costs. Charm Industrial (BiCRS) similarly requires labor-intensive inputting of biomass. Meanwhile, Equatic (Direct Ocean Capture) claims to be low in labor intensity, with a respondent noting that they only “need water and power” and calcium and magnesium, e.g., from synthetic olivine or mining waste.

A fourth dimension was *temporality*. Some options, such as ice protection, afforestation or soil and biomass management (e.g., Charm Industrial or Terraformation or Ecuadorian forestry) as well as seagrass restoration, were believed to be deployable “now.” A Terraformation respondent said they could deploy “right away.” As one Virginian seagrass respondent put it, “this stuff can spread very fast and that’s very different from an estuary or coral reef or even like old growth forest; you cannot restore that habitat in human timescales, and seagrass you can, which makes it pretty special in that regard.” Enhanced weathering (WLIC; Rock flour) can be applied quickly but it remains unclear how durable its effects are, or indeed if they might quickly “peak”. One Glacial Rock Flour respondent observed that, based on one trial, weathering seemed to rather quickly occur (e.g., 2.5–8% over the first three years), before levelling off. In any case, they noted the importance of context and scale: “I think it’s just what are people going to do with it, like, if we applied it on all agricultural land, I think it would have a big impact, not immediately, but over the next couple of decades.” Respondents from the Reef Restoration and Adaptation Program similarly spoke about needing *years* of data to create reliable baselines of coral health, reflecting the complexity of shoreline and ocean ecosystems. Carbon Engineering DAC estimated “five years” until deployment, while Harvard SAI confirmed that 1–6 months is needed just for the indoor experiments to take place, prior to the time needed for any outdoor experiments which would have to follow. Others were not yet ready for outdoor pilots and were still establishing site selection criteria.

*Permanence* of carbon storage, emissions abatement, or temperature reduction are another important aspect of innovation and technical efficacy. Seagrass, along with CarbonCure (carbon storage with cement and concrete applications), and BNB Biochar, were said to provide more or less permanent storage, into the thousands of years, unlike forestry, which could be as short as a decade or even shorter if forests were not properly managed or succumbed to wildfires or pests. As one BNB Biochar respondent staked, “carbon storage is for thousands of years, the biochar stays bound to concrete even if a building is torn down or a road is broken up or replaced, the relevant dust and pebbles keep storing it.” Both Orca DAC and Carbon Engineering DAC spoke about permanent storage as long as carbon reservoirs were properly managed, and Charm Industrial (BiCRS) similarly spoke about storing bio-oil in geological formations such as old oil reservoirs which also are said to have long-term capacity for storage. Temporalities for enhanced weathering and the use of glacial rock flour were more uncertain, respondents spoke about how it would seem to have the potential to be permanent for decades, but there are uncertainties, e.g., how much becomes resident in soils versus washed away in groundwater (and how this affects sequestration) as well as if enhanced weathering quickly hits a plateau in terms of carbon sequestered (i.e., in relation to “yield response curves” and “long-term average weathering rate”), after which point it makes little sense to continue deploying. The durability of Vesta’s storage (marine-based enhanced weathering was also uncertain, and contingent on improved research and understanding of, e.g., processes of ocean circulation and wave dynamics—given that rocks seem to stay in intertidal zone, changes in ocean alkalinity appear to occur in a relatively durable manner but not necessarily to the extent if they were locked away in deep oceans.

Efforts at *scaling up* each experiment also have some interesting similarities and differences, both in terms of the importance of this goal and the variety with which it is pursued. Both reef restoration and seagrass restoration activities are increasingly focused on mechanization and automation to improve the spreading of reef larvae or seeds, to get away from relying on people doing it “by hand”, with a view toward scaling up. Terraformation (reforestation) is seeking to move the opposite way to more people getting involved by hand: “We have also developed a do it yourself seed bank with an instructional guide in order to solve this bottleneck in scaling projects,” one respondent said. Charm Industrial (BiCRS) claims it could easily be deployed at large scale; availability of required biomass (agriculture and forestry residues) currently not seen as a barrier to scaling up. In Direct Ocean Capture, Equatic announced at the time of data collection plans for its first commercial scale facility in Singapore and is pursuing purchase agreements for carbon credits with various companies that it hopes can finance expansion; Captura was at the time of data collection in discussions about using partnerships for scaling with Equinor in Norway, DeepSky in Canada, and other undisclosed parties in Spain and Europe. For them, scaling will happen through a licensing model (i.e., Captura remains an R&D actor mostly): “So Captura will have a licensing business model, which means that we will continue to be an R&D company for the long run as we further develop the Direct Ocean Capture technology and aim to engineer the most promising and effective and low-cost solution in the marine CDR space.”

For WLIC and Glacial Rock flour, a strategy for pilot testing (and eventually scaling up) is to first approach farmers that already use compost, both for logistical reasons (as a means to distribute to material on the field) and because they might be motivated to substitute for costly fertilizers. Those involved with Hawaiian OAE efforts noted that short-term scale up is likely not possible, given high uncertainties and existing policy frameworks: “policymakers are under appreciating the challenges of scaling this up”. They also clearly explicated what could be framed as the “valley of death” from research to commercial scale (something also echoed by Vesta): “This is all great for us in terms of improving our understanding of ocean chemistry and they are great science experiments but if your goal really is to change the world it is a whole big challenge to go from even a reasonably large local field study to sequestering enough carbon from the atmosphere and make any sort of difference.”

The *location* and site of experiments also varied significantly across our 20 cases. Some such as Harvard SAI are indoors only, in labs. Some exist only at small scale pilot sites, such as, Running Tide, and both Orca DAC and Carbon Engineering DAC. Others such as WLIC and glacial rock flour (both enhanced weathering) are simultaneously undertaking small-scale field trials along with, through partnerships with agricultural operators, pursuing tests on actual agricultural operations. Charm Industrial (BiCRS) has a few first and second generation outdoor pyrolizers in place. Vesta (ocean alkalinity enhancement) also falls into this category and confirmed that their experiments are outdoor but at relatively small scale: 600 cubic yards of olivine, which was around 10% of the entire nourishment (regular quartz sand), and spread across 100 yards. Hawaiian OAE is experimenting in controlled environments, using contained mesocosms (not in the open water). By contrast, others such as Virginian Seagrass and Project Seagrass are already widespread outdoors and across various communities. One Virginian Seagrass respondent articulated that theirs is “the largest successful seagrass restoration project by far in the world [at] 9–10 thousand acres; it’s an impressive volume.” Similarly, BNB Biochar and CarbonCure (cement and concrete) are both commercially available throughout the USA and Europe, respectively.

Another aspect of innovation examined was *engagement*. Here, projects ranged from significant to nonexistent involvement of community partners, citizens, and other actors. Engagement was very high for Virginia Seagrass, with engagement ranging from volunteers for seagrass harvesting and outreach and education initiatives at local schools and in local communities (public townhalls, press releases, news articles), along with entering into discussions and avoiding conflicts with, mostly, aquaculture industry and “watermen” (those working clams and oysters). Related to The Nature Conservancy, one of the principal partners, there was also noted to be more emphasis on issues of environmental justice as well as engagement with local minority communities, previously a key shortcoming. Vesta also exhibited strong outreach to local communities, to the extent that the local community voted to install a plaque informing beachgoers about the operations and having annual meetings to report on progress. These were accompanied by regular newsletters providing updates to the nearby community, and engagement with local NGOs and stakeholders (such as the Peconic Estuary Partnership) and local colleges and universities (e.g., Stony Brook University) as well as facilitating a science-focused internship program with local high school students (who visited every Friday during summer). Terraformation (reforestation) reported that “here in Hawaii the community is a big component to everything that we do, right and in particular I think that we are blessed in Hawaii because the local people are so connected to the land.”

Glacial rock flour is an example of intermediate engagement, with ongoing stakeholder discussions in Greenland, as well as partnerships with working farms in three trial countries (the latter is also true for WLIC, though only in the USA). Charm Industrial (BiCRS) similarly reported moderate interaction with farmers and forest managers who provide biomass as well as interaction with local governments and communities at storage sites.

At the other end of the spectrum, Equatic (Direct Ocean Capture) had little discussion of community engagement, mostly highlighting how their transparent way of doing and communicating is aimed to increase credibility and acceptance among regulators and ultimately the public. Captura similarly had minimal public engagement, e.g., some outreach has been through the programs of AltaSea with high school students, but most of their efforts at this stage have been focused on R&D: on “proving that the damn thing works.” Hawaiian OAE respondents also spoke about lack of engagement in this manner: “our project, because it is not a field trial, it is a controlled laboratory experiment, that is why the community is not as involved.”

**Fig. 3:**
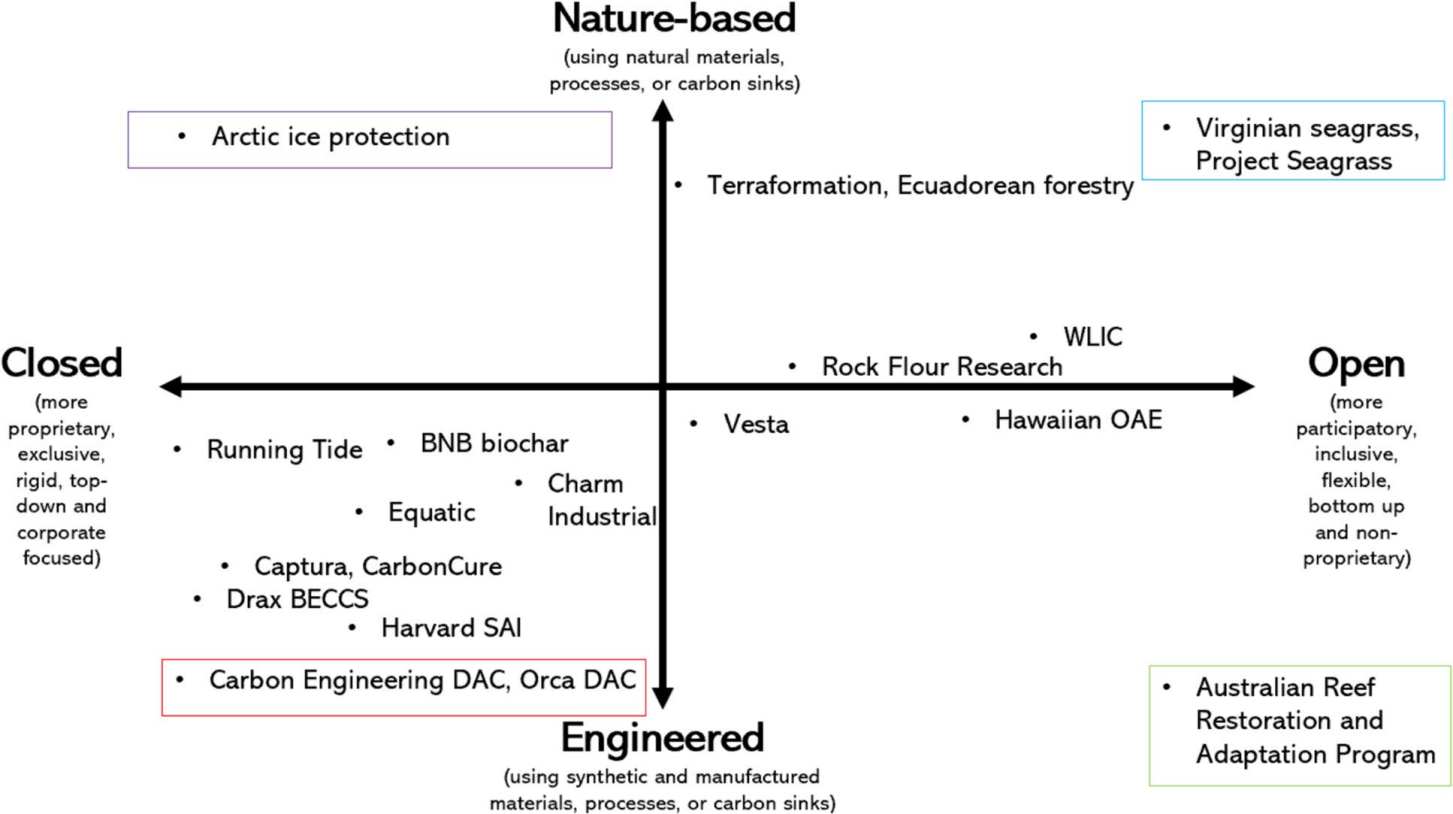
Visualizing the innovation styles of 20 climate intervention experiments. Source: Authors. *DAC* direct air capture, *BECCS* bioenergy with carbon capture and storage, *WLIC* Working Lands Innovation Center, *OAE* ocean alkalinity enhancement, *SAI* stratospheric aerosol injection

A final synthetic aspect of innovation relates to specific innovation *styles* for each case, as to whether they are more bottom-up and open or top-down and closed, more focused on profit and competitive advantage, or on public service and advancing open science. Figure 3 plots these styles across all 20 cases using a typology also grounded in whether approaches are more engineering-oriented or nature-based, as well as whether they are inclusive and non-proprietary, or exclusive and proprietary. Admittedly, this is just one way of visualizing our data and other dimensions include labor vs. technological intensity, conventional vs. novel, or participatory vs. top-down and proprietary, which we have not plotted here due to lack of space.

The diagram shows four examples in each quadrant as extremes. The red box (bottom left) reflects both the Carbon Engineering and Orca DAC projects. These are outliers given their highly engineered focus, energy intensity, emphasis on corporate and proprietary advantage, and their drivers being explicitly profit and private sector interests. In the green box (bottom right), Australia reefs, while also heavily engineering based, given a focus on fogging and shading, genetic enhancement of reef species, and robots dispersing seeds, tend to prioritize open science, with no patents involved and a desire to include tourists, youth, and other citizens. In the purple box (upper left), we see ice protection using the manual removal of natural rubble to change glacial melting. No machines are involved, making it a low-tech intervention with spades and sleds, but it is primarily focused on enhancing tourism experiences, with completely private-sector interest, and no other explicit collaboration. The blue box (upper right) shows nature-based interventions that are highly open, both seagrass projects, involving citizen scientists, free and open sharing of data, and access to sites. Figure 3 also illustrates how most projects fall within a spectrum of this typology and are more hybrid in nature.

### Perceived co-impacts

**Fig. 4:**
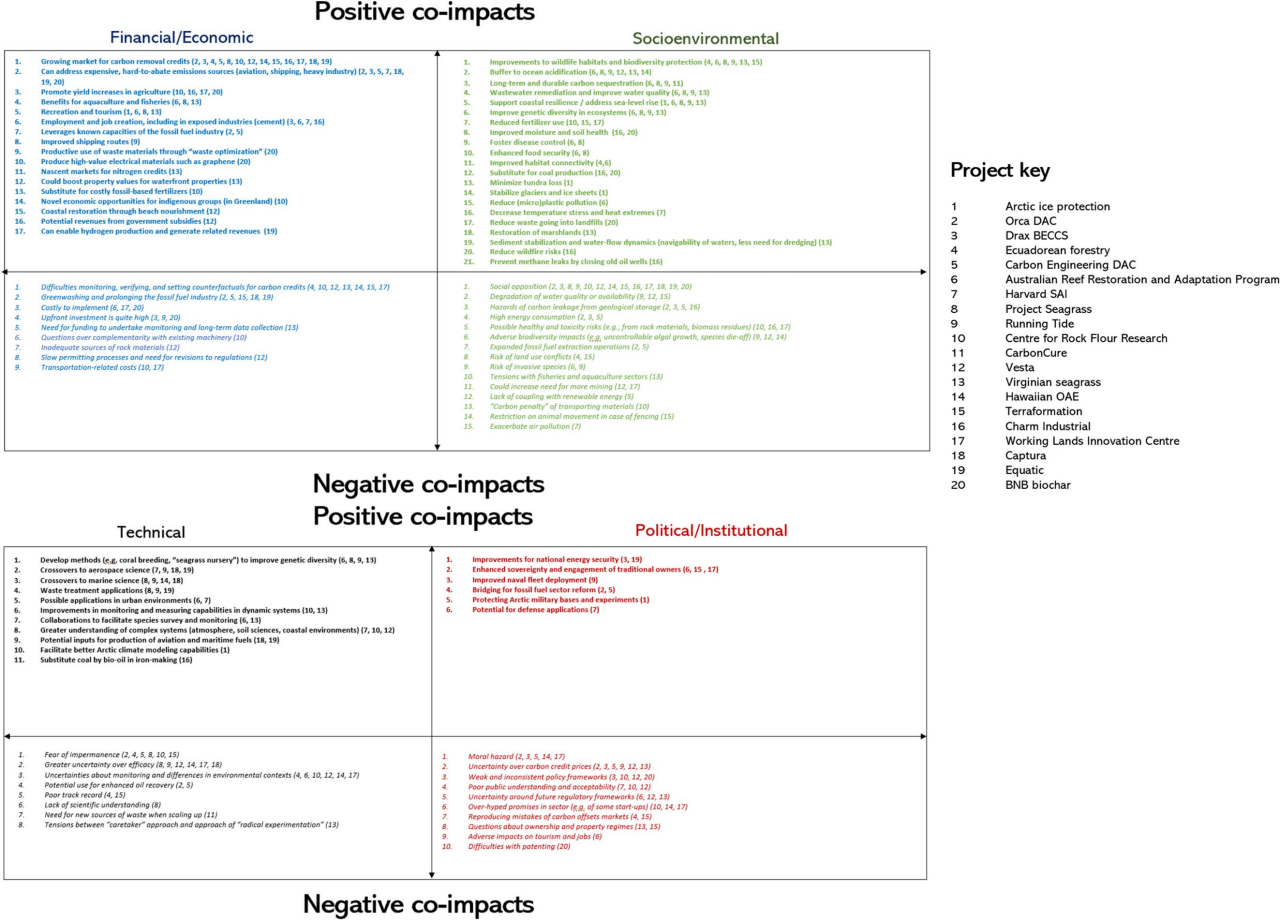
Depicting the 97 perceived co-impacts of 20 global climate and climate intervention experiments. Source: Authors. The numbers in parentheses to any given co-impact refer to the projects mentioned in Table 1, and also summarized by the project key

All 20 cases possess, significant prospective co-impacts from research, deployment and (potential) commercialization, or positive benefits and negative costs and risks. As Fig. 4 presents across all 20 cases, 55 positive co-impacts were identified (in bold text) from our interview data. Of interest, only two of the projects failed to be connected with financial and economic benefits (Arctic ice protection, Harvard SAI). More than half of the projects were believed to have significant economic and financial potential to capture a growing market for carbon credits, or to contribute to new markets for carbon removal or blue carbon credits. Many projects were also praised by respondents for positive socioenvironmental co-impacts such as creating or improving wildlife habitats and protecting biodiversity (Ecuadorean forestry, the Reef Restoration and Adaptation Program, Running Tide, Terraformation, Project Seagrass, Virginian Seagrass), enhancing food security (the Reef Restoration and Adaptation Program, Project Seagrass), and reducing need for fertilizers (Rock Flour Research, WLIC). Technical co-impacts were less mentioned overall, but included cross applications within cities and urban environments (the Reef Restoration and Adaptation Program, Harvard SAI) or crossovers to other sectors such as energy production (Equatic) and scientific domains, notably for aerospace and marine sciences (Harvard SAI, Project Seagrass, Running Tide, Captura, Equatic), along with improving understanding of complex systems such as the soil sciences or coastal environments (Vesta, Rock Flour Research, Virginian Seagrass) and facilitating better climate modeling capabilities in the Arctic (Arctic ice protection).

Of note, political and institutional benefits received the least attention, with only a few projects noting potential improvements for energy security (Drax BECCS, Equatic) or greater recognition of Indigenous sovereignty and traditional landowners (Reef Restoration and Adaptation Program, WLIC). The sectoral incidence of these positive co-benefits varies: SAI and the fogging and shading aspects of the Reef Restoration and Adaptation Program and parts of Running Tide’s approach would have strong linkages to the aerospace and military industries; blue carbon interventions have the potential to benefit fisheries and coastal communities; Ecuadorian forestry and Terraformation would have benefits for forest communities; enhanced rock weathering is strongly tied to and benefit farming and agricultural communities; both DAC experiments have strong linkages to the oil and gas industry.

However, as Fig. 4 indicates, 42 negative co-impacts (in italics) were also identified. Prospective difficulties with monitoring, reporting, and verifying of carbon (removal) credits were mentioned most frequently as economic and financial risks (e.g., Ecuadoran forestry, Rock Flour Research, Vesta, Hawaiian OAE), closely followed by risks—identified by the authors—of greenwashing and prolonging the fossil fuel industry (e.g., Orca DAC, Carbon Engineering DAC). Lack of social acceptance (relevant for two-thirds of the projects) and degradation of water quality and availability (e.g., Running Tide, Vesta, Terraformation) were seen as serious socioenvironmental risks. One respondent from Harvard SAI captured this lack of acceptance well, noting that “some of these experiments sound totally insane, they are really out there, in the eyes of most people, it’s [geoengineering] like using opioids, it doesn’t address the problem, and you need to use them more and more in increasing amounts to meet your needs.” Another respondent from Running Tide added that “I do worry that it’s a smokescreen to some extent. I probably worry more about that with direct air capture, the engineering solutions.” Fears of the impermanence of storage and sequestration (e.g., Carbon Engineering DAC, Terraformation, Project Seagrass, Rock Flour Research) and uncertainty over efficacy (e.g., Project Seagrass, Running Tide, Hawaiian OAE, WLIC) were posed as recurring technical risks, while moral hazard (e.g., Orca DAC, Drax BECCS), uncertainty over carbon pricing (e.g., Running Tide, Virginia Seagrass, Carbon Engineering DAC, Equatic, Captura), and weak and inconsistent policy and regulatory frameworks (Rock Flour Research, Vesta, BNB Biochar) were evident as institutional and political risks. As one respondent from the Reef Restoration and Adaptation Program added, the scale of these risks will directly match the degree to which the technologies are used potentially growing with the scale of deployment: “negative side effects scale with the amounts you use, larger amounts create larger serious consequences.” A BNB Biochar expert also cautioned against going too far the other way, toward “certification hell” where permits and red tape slow everything down until it is too late to contribute to tackling climate change.

Other respondents spoke about how this tension of risks vs. benefits—of the total 97 distinct co-impacts involved in potential research, deployment, and commercialization of these approaches—is exactly what makes discussions of climate intervention so fraught. Looking at Fig. 4, we can identify different underlying patterns to such tensions, depending on the type of co-impact. The socioenvironmental dimension is notable for having both the most positive and negative co-impacts identified (by interviewees) of any of the dimensions, with a relative edge to positive co-impacts here. Positive co-impacts of a financial and economic nature were also prominent, with a much greater disparity with negative co-impacts. While technical co-impacts were less mentioned overall, we again identify a broad balance between positive and negative aspects. Conversely, political and institutional co-impacts were disproportionately negative in tone. One supposition here could be that, given that our interviewees were predominantly researchers and/or proponents of the methods, their attention to such impacts is more limited—and indeed might be over-focused on how it (negatively) impacts their own work. Nonetheless, this finding does echo the relative lack of attention to political and institutional factors in the climate intervention domain more broadly (e.g., McLaren and Corry 2021; Möller 2020; Sovacool et al. 2023b).

Returning to the tensions between risks and benefits, this is also broadly illustrative of why the need for further research and understanding is so necessary, not least given how the ways in which these risks and benefits materialize could be context dependent (NASEM 2019, 2021; Bellamy et al. 2012; Sovacool et al. 2023b). At the same time, it could be that some risks (and benefits) are qualitatively different in nature, for instance in their risks of provoking catastrophe (e.g., Hartzell-Nichols 2012; Smith 2018)—for some, they are thus grounds for proceeding with strict precaution (Hartzell-Nichols 2012; McKinnon 2019). In any case, many of the interviewees pointed to this tension as a reason why the international community, including engagement with those who would be directly affected, needs a more inclusive, and holistic, dialogue (Jinnah et al. 2018; Hourdequin 2019; Whyte 2012; Morrow 2020). As one Harvard SAI interviewee explained: “We urgently need an intelligent, reasonable global conversation about carbon removal and geoengineering, taking stock of the options we have, with all of their drawbacks and benefits, not as stepchild or ugly duckling, an assessment of where we are, to assess interventions in a mature way, to properly weigh the full gamut of information on views and risks.”

## Conclusion

Climate intervention research, experiments, and pilots involve a diversity of actor coalitions, propose solutions to a plethora of emergent scientific and environmental problems, and are increasingly coalescing around manifold requirements about how those technologies will perform into the future. Salient characteristics of their innovation dynamics and styles, as revealed by our 20 case studies and respondent interviews along with ethnographic observations, exemplify immense heterogeneity across attributes as diverse as efficacy, cost, scale, location, permanence, and temporality.

In response to a challenge as complex, multi-scalar, and multifarious as climate change, this reveals how the search for potential solutions broadly mirrors the complexity of the problem itself. More skeptically, it might also be that the amount of resources flowing to addressing this problem, and its diverse impacts, is attracting attention broadly across the sectors of economy and society. In any case, perceptions of the different experiments reveal notable tensions between risks and benefits, with almost one hundred different perceived co-impacts evident, involving an array of distinct financial and economic, socioenvironmental, technical, and institutional and political dimensions. Co-impacts of a socioenvironmental and financial/economic nature are notably more commonly identified, especially in contrast to institutional and political aspects. Given the broad consensus of the governance challenges of climate intervention approaches (e.g., NASEM 2019, 2021; Honegger et al. 2022; Reynolds 2019; Gupta et al. 2020; McLaren and Corry 2021), this underscores a possible neglect of such aspects among those planning and undertaking the experiments in question.

The utility of climate interventions involving CDR and solar radiation modification depends not necessarily on the technology, but on how they are managed, on the actor coalitions that support or oppose them, on the innovation dynamics and styles evident, or the perceived co-impacts at play. There is some agreement that innovations and experiments have the potential to work well, when undertaken with proper safeguards in place, properly regulated and enforced, done in a fully transparent manner, with requisite accountability, and with robust measuring and monitoring of environmental impacts and meaningful engagement with local communities (e.g., Boettcher et al. 2023; Gardiner and Fragnière 2020; Hubert 2021; Nawaz and Lezaun 2024; Nawaz et al. 2024; Reynolds 2019).

However, when environmental impacts are poorly managed, when promises are overhyped, when community well-being or social attitudes are downplayed or disregarded, uncertainty can give rise to public opposition and, in extreme cases, a call for moratoria or sustained protest. In this way, any purported benefits of climate interventions can be as much a mirage as a miracle; they can quickly evaporate under the wrong sociotechnical, innovation, or political conditions.

## Data Availability

The data used is confidential and cannot be shared.

## References

[CR1] Acemoglu D, Bimpikis K, Ozdaglar A (2011) Experimentation, patents, and innovation. Am Econ J Microecon 3(1):37–77

[CR2] Aldy JE, Felgenhauer T, Pizer WA, Tavoni M, Belaia M, Borsuk ME et al (2021) Social science research to inform solar geoengineering. Science 374(6569):815–818 34762479 10.1126/science.abj6517

[CR3] Anderson K, Peters G (2016) The trouble with negative emissions. Science 354(6309):182–183 27738161 10.1126/science.aah4567

[CR4] Angrosino MV (2007) Naturalistic observation, 1st edn. Routledge. 10.4324/9781315423616

[CR5] Babiker M, Berndes G, Blok K, Cohen B, Cowie A, Geden O, Ginzburg V, Leip A, Smith P, Sugiyama M, Yamba F (2022) Cross-sectoral perspectives supplementary material. In: Shukla PR, Skea J, Slade R, Al Khourdajie A, van Diemen R, McCollum D, Pathak M, Some S, Vyas P, Fradera R, Belkacemi M, Hasija A, Lisboa G, Luz S, Malley J (eds) IPCC, 2022: climate change 2022: mitigation of climate change. Contribution of Working Group III to the Sixth Assessment Report of the Intergovernmental Panel on Climate Change

[CR01] Baiman R, Clarke S, Elsworth C, Field L, MacCracken M, Macdonald J, Mitchell D, Oeste FD, Reed S, Salter S, Simmens H, Tao Y, Tulip R (2024) Addressing the urgent need for direct climate cooling: Rationale and options. Oxford Open Clim Change 4(1):kgae014

[CR6] Bakker S (2011) Harro Van Lente Marius Meeus, “Arenas of expectations for hydrogen technologies.” Technol Forecast Soc Change 78:152–162

[CR02] Baresi U, Baum CM, Fischer TB, Lockie S, Piggott-McKellar A, Graham V, Bohensky E, Fritz LB, Shumway N, Harrison DP, Foster R, Sovacool BK, Vella K, Ristovski Z (2025) A call for strategic assessments of regional applications of solar radiation management: Exploring the challenges and opportunities from marine cloud brightening and albedo surface modification. Environ Impact Assess Rev 110:107701, pp. 1–16

[CR7] Battersby F, Heap RJ, Gray AC, Workman M, Strivens F (2022) The role of corporates in governing carbon dioxide removal: outlining a research agenda. Front Clim 4:686762. 10.3389/fclim.2022.686762

[CR8] Baum CM, Low S, Sovacool BK (2023) Coupling for climate intervention: sectoral and sustainability couplings for carbon removal and solar geoengineering pathways. Technol Forecast Soc Change 194:122734

[CR9] Baum CM, Fritz L, Low S, Sovacool BK (2024) Public perceptions and support of climate intervention technologies across the Global North and Global South. Nat Commun 15(1):2060 38448460 10.1038/s41467-024-46341-5 PMC10918186

[CR10] Bellamy R, Chilvers J, Vaughan NE, Lenton TM (2012) A review of climate geoengineering appraisals. Wiley Interdiscip Rev Clim Change 3(6):597–615

[CR11] Boettcher M, Chai F, Canothan M, Cooley S, Keller DP, Klinsky S, Lezaun J, Renforth P, Scobie M, Webb RM (2023) A code of conduct for marine carbon dioxide removal research [Report]. Aspen Institute, Energy & Environment Program. https://www.aspeninstitute.org/publications/a-code-of-conduct-for-marine-carbon-dioxide-removal-research/

[CR12] Buck HJ et al (2023) Why residual emissions matter right now. Nat Clim Change 13(4):351–358

[CR13] Bukirwa P, Chathurika G, McClellan M, Sheerazi HA, Westler G (2024) Delivering equitable and meaningful community benefits via clean hydrogen hubs. Rocky Mountain Institute, Basalt

[CR14] Burgess-limerick T, Burgess-limerick R (1998) Conversational interviews and multiple-case research in psychology. Aust J Psychol 50(2):63–70

[CR15] Burke J, Gambhir A (2022) Policy incentives for greenhouse gas removal techniques: the risks of premature inclusion in carbon markets and the need for a multi-pronged policy framework. Energy Clim Change 3:100074

[CR16] Carey AL, Rentscher KE, Mehl MR (2020) Naturalistic observation of social interactions. In: The Wiley encyclopedia of health psychology, pp 373–383

[CR17] Carton W et al (2020) Negative emissions and the long history of carbon removal. Wiley Interdiscip Rev Clim Change 11(6):e671

[CR18] Chiquier S, Patrizio P, Bui M, Sunny N, Mac Dowell N (2022) A comparative analysis of the efficiency, timing, and permanence of CO_2_ removal pathways. Energy Environ Sci 15(10):4389–4403

[CR19] Christiansen KL, Hajdu F, Mollaoglu EP, Andrews A, Carton W, Fischer K (2023) “Our burgers eat carbon”: investigating the discourses of corporate net-zero commitments. Environ Sci Policy 142:79–88

[CR20] Cole AJ, Mata L, Paul NA, de Nys R (2014) Using CO_2_ to enhance carbon capture and biomass applications of freshwater macroalgae. GCB Bioenergy 6:637–645. 10.1111/gcbb.12097

[CR21] Cooley SR et al (2023) Sociotechnical considerations about ocean carbon dioxide removal. Annu Rev Mar Sci 15:41–66 10.1146/annurev-marine-032122-113850 35850491

[CR22] Deuten JJ, Rip A (2000) The narrative shaping of a product creation process. In: Brown N, Rappert B, Webster A (eds) Contested futures: a sociology of prospective techno-science. Aldershot, Ashgate, Burlington, pp 65–86

[CR23] Field CB, Mach KJ (2017) Rightsizing carbon dioxide removal. Science 356(6339):706–707 28522498 10.1126/science.aam9726

[CR24] Fieldman G (2011) Neoliberalism, the production of vulnerability and the hobbled state: Systemic barriers to climate adaptation. Clim Dev 3(2):159–174

[CR25] Fritz L, Baum CM, Low S et al (2024) Public engagement for inclusive and sustainable governance of climate interventions. Nat Commun 15:4168. 10.1038/s41467-024-48510-y 38755215 10.1038/s41467-024-48510-y PMC11099155

[CR26] Frumhoff PC, Stephens JC (2018) Towards legitimacy of the solar geoengineering research enterprise. Philos Trans R Soc A Math Phys Eng Sci 376(2119):20160459 10.1098/rsta.2016.0459 PMC5897829 29610369

[CR27] Gamble C, Debney A, Glover A, Bertelli C, Green B, Hendy I, Lilley R, Nuuttila H, Potouroglou M, Ragazzola F, Unsworth R, Preston J (2021) Seagrass restoration handbook. Zoological Society of London, London

[CR28] Gardiner SM, Fragnière A (2020) The tollgate principles for the governance of geoengineering: moving beyond the Oxford principles to an ethically more robust approach. In: Gardiner SM, McKinnon C, Fragnière A (eds) The ethics of “geoengineering” the global climate. Routledge, London, pp 9–40

[CR29] Giannousakis A et al (2021) How uncertainty in technology costs and carbon dioxide removal availability affect climate mitigation pathways. Energy 216:119253

[CR30] Gupta A, Möller I, Biermann F, Jinnah S, Kashwan P, Mathur V, Nicholson S (2020) Anticipatory governance of solar geoengineering: conflicting visions of the future and their links to governance proposals. Curr Opin Environ Sustain 45:10–19 32843906 10.1016/j.cosust.2020.06.004 PMC7423514

[CR31] Hartzell-Nichols L (2012) Precaution and solar radiation management. Ethics Policy Environ 15(2):158–171

[CR32] Hauser OP, Linos E, Rogers T (2017) Innovation with field experiments: studying organizational behaviors in actual organizations. Res Organ Behav 37:185–198

[CR33] Herzog H, Caldeira K, Reilly J (2003) An issue of permanence: assessing the effectiveness of temporary carbon storage. Clim Change 59(3):293–310

[CR34] Hilser H, Hiraldo L, Moreau C, Draiby A, Cox E, Andrews MG, Winks L, Walworth NG (2024) Public engagement and collaboration for carbon dioxide removal: lessons from a project in the Dominican Republic. Front Clim 6:1290999

[CR35] Honegger M, Baatz C, Eberenz S, Holland-Cunz A, Michaelowa A, Pokorny B, Winkler M (2022) The ABC of governance principles for carbon dioxide removal policy. Front Clim 4:884163

[CR36] Hourdequin M (2019) Geoengineering justice: the role of recognition. Sci Technol Human Values 44(3):448–477. 10.1177/0162243918802893

[CR37] Hubert AM (2021) A code of conduct for responsible geoengineering research. Glob Policy 12:82–96

[CR38] Irvine PJ, Kravitz B, Lawrence MG, Muri H (2016) An overview of the Earth system science of solar geoengineering. Wiley Interdiscip Rev Clim Change 7(6):815–833

[CR39] Jinnah S, Nicholson S, Flegal J (2018) Toward legitimate governance of solar geoengineering research: a role for sub-state actors. Ethics Policy Environ 21(3):362–381

[CR40] Kindon S, Pain R, Kesby M (2007) Participatory action research approaches and methods. Connecting people, participation and place. Routledge, Abingdon

[CR41] Kivimaa P, Hildén M, Huitema D, Jordan A, Newig J (2017) Experiments in climate governance—a systematic review of research on energy and built environment transitions. J Clean Prod 169:17–29

[CR42] Kremer M (2020) Experimentation, innovation, and economics. Am Econ Rev 110(7):1974–1994

[CR43] Low S, Baum CM, Sovacool BK (2022) Taking it outside: exploring social opposition to 21 early-stage experiments in radical climate interventions. Energy Res Soc Sci 90:102594

[CR44] Low S, Fritz L, Baum CM, Sovacool BK (2024) Public perceptions on carbon removal from focus groups in 22 countries. Nat Commun 15(1):3453 38658623 10.1038/s41467-024-47853-w PMC11043362

[CR45] MacDonald C (2012) Understanding participatory action research: a qualitative research methodology option. Can J Act Res 13(2):34–50

[CR46] MacMartin DG, Visioni D, Kravitz B, Richter JH, Felgenhauer T, Lee WR, Morrow DR, Parson EA, Sugiyama M (2022) Scenarios for modeling solar radiation modification. Proc Natl Acad Sci 119(33):e2202230119 35939702 10.1073/pnas.2202230119 PMC9388149

[CR47] Mahony M, Hulme M (2018) Epistemic geographies of climate change: science, space and politics. Prog Hum Geogr 42(3):395–424

[CR48] Maliniak D, Parajon E, Powers R (2021) Epistemic communities and public support for the Paris agreement on climate change. Polit Res Q 74(4):866–881

[CR49] Martiskainen MS, Axon BK, Sovacool SS, Rio DFD, Axon K (2020) Contextualizing climate justice activism: knowledge, emotions, motivations, and actions among climate strikers in six cities. Glob Environ Change 65:102180

[CR50] McKinnon C (2019) Sleepwalking into lock-in? Avoiding wrongs to future people in the governance of solar radiation management research. Environ Polit 28(3):441–459

[CR51] McLaren D, Corry O (2021) The politics and governance of research into solar geoengineering. Wiley Interdiscip Rev Clim Change 12(3):e707

[CR52] Möller I (2020) Political perspectives on geoengineering: navigating problem definition and institutional fit. Glob Environ Polit 20(2):57–82. 10.1162/glep_a_00547

[CR53] Morrison TH, Adger WN, Agrawal A et al (2022) Radical interventions for climate-impacted systems. Nat Clim Change 12:1100–1106. 10.1038/s41558-022-01542-y

[CR54] Morrison TH, Barnett J, Gurney GG, Lau J, Barnes ML, Cinner J, Hettiarachchi M, Cohen P (2024) Overcoming lock-in of science-policy responses to reef heating. Mar Policy 170:106380

[CR55] Morrow DR (2020) A mission-driven research program on solar geoengineering could promote justice and legitimacy. In: The ethics of “geoengineering” the global climate. Routledge, pp 207–229

[CR56] NASEM (2019) Negative emissions technologies and reliable sequestration: a research agenda. National Academies of Sciences, Engineering, and Medicine (NASEM), Washington DC. 10.17226/25259 31120708

[CR57] NASEM (2021) Reflecting sunlight: recommendations for solar geoengineering research and research governance. National Academies of Sciences, Engineering, and Medicine (NASEM), Washington DC. 10.17226/25762

[CR58] Nawaz S, Lezaun J (2024) Grappling with a sea change: tensions in expert imaginaries of marine carbon dioxide removal. Glob Environ Change 85:102806

[CR59] Nawaz S, Scott-Buechler C, Caggiano H (2024) An independent public engagement body is needed to responsibly scale carbon removal in the US. Environ Res Lett 19(1):011002

[CR60] Oberlack C, Boillat S, Brönnimann S, Gerber JD, Heinimann A, Speranza CI, Messerli P, Rist S, Wiesmann U (2018) Polycentric governance in telecoupled resource systems. Ecol Soc 23(1)

[CR61] Oksanen A-A (2023) Dimming the midnight sun? Implications of the Sámi Council’s intervention against the SCoPEx project. Front Clim. 10.3389/fclim.2023.994193

[CR62] Peters M, Fudge S, Hoffman SM, High-Pippert A (2012) Carbon management, local governance and community engagement. Carbon Manag 3(4):357–368

[CR63] Pierce JJ, Osei-Kojo A (2022) 32. The advocacy coalition framework. In: Handbook on theories of governance, p 353

[CR64] Reynolds JL (2019) Solar geoengineering to reduce climate change: a review of governance proposals. Proc R Soc A 475(2229):20190255 31611719 10.1098/rspa.2019.0255 PMC6784395

[CR65] Ruseva T et al (2020) Rethinking standards of permanence for terrestrial and coastal carbon: implications for governance and sustainability. Curr Opin Environ Sustain 45:69–77

[CR66] SAPEA (2024) Solar radiation modification. SAPEA, Berlin.10.5281/zenodo.14283096

[CR67] Smith PT (2018) Legitimacy and non-domination in solar radiation management research. Ethics Policy Environ 21(3):341–361. 10.1080/21550085.2018.1562528

[CR68] Sovacool BK, Hess DJ (2017) Ordering theories: typologies and conceptual frameworks for sociotechnical change. Soc Stud Sci 47(5):703–750 28641502 10.1177/0306312717709363 PMC5648049

[CR69] Sovacool BK, Baum CM, Low S (2022) Determining our climate policy future: expert opinions about negative emissions and solar radiation management pathways. Mitig Adapt Strat Glob Change 27(8):58 10.1007/s11027-022-10030-9 PMC9527724 36200076

[CR70] Sovacool BK, Baum CM, Low S, Fritz L (2023a) Coral reefs, cloud forests and radical climate interventions in Australia’s Wet Tropics and Great Barrier Reef. PLOS Clim 2(10):e0000221

[CR71] Sovacool BK, Baum CM, Low S (2023b) Beyond climate stabilization: exploring the perceived sociotechnical co-impacts of carbon removal and solar geoengineering. Ecol Econ 204:107648

[CR72] Sovacool BK, Iskandarova M, Hall J (2023c) Industrializing theories: a thematic analysis of conceptual frameworks and typologies for industrial sociotechnical change in a low-carbon future. Energy Res Soc Sci 97:102954

[CR73] Sovacool BK, Iskandarova M, Geels FW (2023d) “Bigger than government”: exploring the social construction and contestation of net-zero industrial megaprojects in England. Technol Forecast Soc Change 188(2023):122332

[CR74] Sovacool BK, Baum CM, Cantoni R, Low S (2024a) Actors, legitimacy, and governance challenges facing negative emissions and solar geoengineering technologies. Environ Polit 33(2):340–365. 10.1080/09644016.2023.2210464 10.1080/09644016.2023.2210464 PMC10911681 38444630

[CR03] Sovacool BK, Baum CM, Low S, Fritz L (2024b) The sociotechnical dynamics of blue carbon management: Testing typologies of ideographs, innovation, and co-impacts for marine carbon removal. Environ Sci Policy 155(2024):103730

[CR75] Stoddard I et al (2021) Three decades of climate mitigation: why haven’t we bent the global emissions curve? Annu Rev Environ Resour 46:653–689

[CR76] Tall A, Lynagh S, Blanco Vecchi C, Bardouille P, Montoya Pino F, Shabahat E et al (2021) Enabling private investment in climate adaptation and resilience: current status, barriers to investment and blueprint for action

[CR77] Thomke SH (2003) Experimentation matters: unlocking the potential of new technologies for innovation. Harvard Business Press, Brighton

[CR78] United Nations Environment Program (2020) Out of the blue: the value of seagrasses to the environment and to people. UNEP, Nairobi

[CR79] Ürge-Vorsatz D et al (2014) Measuring the co-benefits of climate change mitigation. Annu Rev Environ Resour 39:549–582

[CR80] van Enk AAJ (2009) The shaping effects of the conversational interview: an examination using Bakhtin’s theory of genre. Qual Inq 15(7):1265–1286

[CR81] Van Lente H (1993) Promising technologies: the dynamics of expectations in technological developments. Twente University, Enschede

[CR82] Wang S, Bai X (2022) Compatibility in cross-city innovation transfer: Importance of existing local experiments. Environ Innov Soc Trans 45:52–71

[CR83] Weible CM, Ingold K, Nohrstedt D, Henry AD, Jenkins-Smith HC (2020) Sharpening advocacy coalitions. Policy Stud J 48(4):1054–1081

[CR84] Whyte KP (2012) Now this! Indigenous sovereignty, political obliviousness and governance models for SRM research. Ethics Policy Environ 15(2):172–187. 10.1080/21550085.2012.685570

[CR85] Wilson C et al (2020) Granular technologies to accelerate decarbonization. Science 368(6486):36–39 32241941 10.1126/science.aaz8060

[CR86] Zhao M, Cao L, Visioni D, MacMartin DG (2024) Carbon cycle response to stratospheric aerosol injection with multiple temperature stabilization targets and strategies. Earth’s Future 12:e2024EF004474. 10.1029/2024EF004474

